# Breast cancer teams: the impact of constitution, new cancer workload, and methods of operation on their effectiveness

**DOI:** 10.1038/sj.bjc.6601073

**Published:** 2003-07-01

**Authors:** R Haward, Z Amir, C Borrill, J Dawson, J Scully, M West, R Sainsbury

**Affiliations:** 1 Arthington House, Hospital Lane, Cookridge Hospital, Leeds LS16 6QB, UK; 2 Epidemiology and Health Services Research, University of Leeds, UK; 3 Aston Business School, University of Aston, UK; 4 Department of Surgery, Royal Free University College Medical School, UK

**Keywords:** breast cancer, clinical nurse specialist, collaboration, leadership, multidisciplinary team, outcome and process assessment (health care), workload, caseload

## Abstract

National guidance and clinical guidelines recommended multidisciplinary teams (MDTs) for cancer services in order to bring specialists in relevant disciplines together, ensure clinical decisions are fully informed, and to coordinate care effectively. However, the effectiveness of cancer teams was not previously evaluated systematically. A random sample of 72 breast cancer teams in England was studied (548 members in six core disciplines), stratified by region and caseload. Information about team constitution, processes, effectiveness, clinical performance, and members' mental well-being was gathered using appropriate instruments. Two input variables, team workload (*P*=0.009) and the proportion of breast care nurses (*P*=0.003), positively predicted overall clinical performance in multivariate analysis using a two-stage regression model. There were significant correlations between individual team inputs, team composition variables, and clinical performance. Some disciplines consistently perceived their team's effectiveness differently from the mean. Teams with shared leadership of their clinical decision-making were most effective. The mental well-being of team members appeared significantly better than in previous studies of cancer clinicians, the NHS, and the general population. This study established that team composition, working methods, and workloads are related to measures of effectiveness, including the quality of clinical care.

The last decade saw substantial changes in cancer care in the United Kingdom (UK). New health policies, notably Calman-Hine (Department of Health, 1995) and the National Cancer Plan (Department of Health, 2000), were introduced, supported by detailed guidelines for breast (BASO, 1995) and other cancers. The Improving Outcomes Guidance (IOG) series started with breast cancer in 1996. It recommended that all breast patients should be referred to multidisciplinary teams (MDTs) managing a minimum of 100 new breast cancer patients per year. Expert opinion (Working Party of the British Breast Group, 1995) also recommended the establishment of specialist MDTs for breast cancer.

These initiatives were stimulated by concerns about comparative outcomes (Berino *et al*, 1995, 1999), and by the variability of cancer care. Observational studies (Chouillet *et al*, 1994; Sainsbury *et al*, 1995b) showed considerable variability in breast service delivery. Greater specialisation (Gillis and Hole, 1996) and higher caseloads (Sainsbury *et al*, 1995a) were both shown to be associated with survival benefits.

The successful implementation of breast screening (Forrest, 1986) over the period 1989–1991 exposed a contrast between well-planned arrangements for screen-detected disease and the variability of symptomatic services. Prior to screening, typical services for symptomatic breast patients were provided by general surgeons (Link, 2000), usually within general surgical outpatient clinics. Many surgeons treating symptomatic patients were not recognised as having a special interest in breast conditions. Decisions on management seldom involved consultants in other disciplines. The involvement of oncologists depended on referrals by surgeons.

Over the last decade, this model has largely been replaced by:

- Individual specialisation in breast disease.
- Multiprofessional working based on multidisciplinary breast teams.
- Increasing demarcation of breast services, with designated clinics and facilities.

While these changes predated screening in a few locations, most hospitals implemented them after publication of Calman-Hine in 1995 and the IOG in 1996. A nonrecurring allocation of £10 million was provided in England (NHS Executive, 1997) to stimulate the process.

Team working is important in health-care delivery. Primary care team working has been reported (Wood *et al*, 1994) to improve health-care delivery and staff motivation, giving better detection, treatment, follow-up, and outcome in hypertension (Adorian *et al*, 1990). Team working improved patients' access to primary care (Marsh, 1991) and the deployment of skills and expertise (Marsh, 1991; Hasler, 1994; Bradley, 1996), producing more cost-effective services (Marsh, 1991). A study (Jansson *et al*, 1992) monitoring patient contacts for 6 years after the introduction of team working attributed improved patient access to primary care to greater accessibility and continuity provided by teams. The benefits of multidisciplinary treatment in advanced colorectal cancer were related primarily to the access to, and use of, standardised and up-to-date therapy (Landheer *et al*, 2001), the first published study to examine the relationship between cancer teams and the quality of care.

A number of elements are crucial to effective team working (Guzzo and Shea, 1992). Individuals should feel their work is essential to the team, their roles should be meaningful and rewarding, and their contributions should be identifiable. Teams should have intrinsically interesting tasks to perform, clear, shared objectives, and feedback on whether they achieved them. Team leader decisions and behaviours influence team effectiveness (Tannenbaum *et al*, 1996). Leaders who listened to members and incorporated their ideas improved team decisions (Norrgren and Schaller, 1999). When teams have autonomy to determine their working procedures, this can reduce costs (Kirkhart, 1995). Research on workplace teams has been dominated by the theoretical approach (West *et al*, 1998) summarised in Figure 1, to

**Figure 1:**
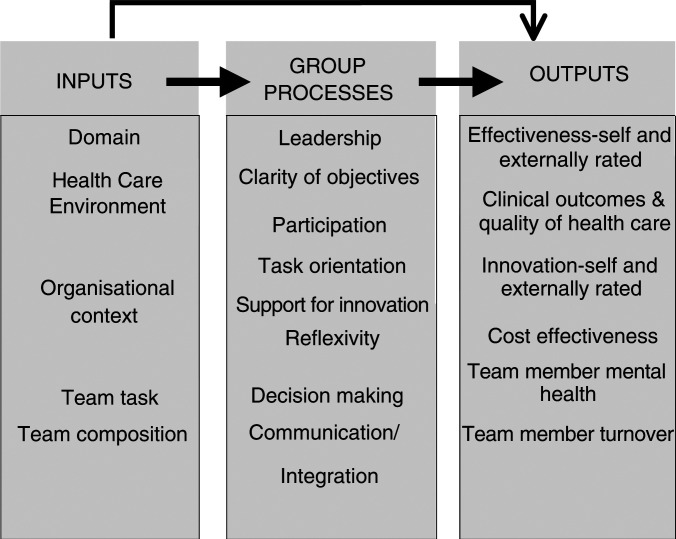
Input, process, and output model of team effectiveness.

examine relationships between team inputs, how teams work together, and measures of outcome. This model underpinned the following study of breast teams, which evaluated a large random sample of breast teams in England.

## MATERIALS AND METHODS

### Sample teams

The pragmatic recruitment target was half the breast teams in England (95 teams). Allowing for nonresponders, a sample of 113 teams was randomly selected from the 190 breast teams listed in the Cancer Relief Macmillan Directory (The Macmillan Directory, 1996). The number in each region represented a constant sampling proportion, that is, the proportion in that region, relative to England. Teams were stratified within regions by their annual new cancer caseloads, with half drawn above and below the regional mean. Questionnaire data were collected over 12 months from the middle of 1999 to 2000.

Invitations to participate were sent to lead breast clinicians. Teams that agreed to participate nominated their preferred contact person, who completed the first of three questionnaires. This provided basic information including the names of members in core disciplines (COG, 1996) (breast surgeons, breast nurses, clinical and medical oncologists, histopathologists, and radiologists). Data on team functioning and self-reported effectiveness were collected through confidential personal questionnaires returned individually to the researchers. The nominated contact provided the clinical data for their team. All research tools were piloted and revised before use.

### Measures used

These covered inputs, process, and outcomes. Information on team inputs was obtained from the basic team questionnaire and from individual questionnaires. It included both data about the whole team and individual members, including:

- The team itself: work locations, meetings, history, and chronology.
- Membership: number and range of professionals in the team.
- Individual involvement: professional background, grade, training, experience, time commitment and duration of team membership, and tenure in the Trust.

Team process was assessed for seven categories of team working. Four were from the Team Climate Inventory (TCI) (West and Anderson, 1996), an established measure based on the theoretical model (West *et al*, 1998). These covered team participation, clarity of and commitment to team objectives, emphasis on quality, and support for innovation. Three further dimensions were included to test a greater range of team functioning. These were: reflexivity (West, 1996), the extent to which team members reflected upon objectives, strategies and processes and made changes accordingly; team innovation, the extent to which the team introduced innovations in objectives, work strategies, processes, and relationships; and leadership, identifying who were the leaders.

Team Outcomes were assessed in three ways; self-reported effectiveness, clinical performance, and mental well-being.

Self-reported effectiveness was derived from the individual questionnaires in which each core member rated their team across a range of dimensions. These were developed for this study using a stakeholder analysis. This began with a workshop of invited representatives from all relevant clinical disciplines, commissioners, managers, and patients. They identified 31 measures that were aggregated into eight effectiveness groups by factor analysis:

- Accessibility of service, communication with patients.
- Accuracy and timing of diagnosis.
- Enabling informed patient choices, maintaining confidentiality.
- Provision of psychosocial support.
- Primary treatment provided.
- Communication within the team.
- Auditing practice and involvement in research.
- Efficient use of resources.

Expert advice and documented evidence were used to identify suitable markers to assess teams' clinical performance, with at least one measure for each key area of activity. These data were collected for 1 year's activity of the team, either the calendar year 1998, or the financial year 1998/1999. This was at the discretion of teams, depending on data availability. The measures were kept as simple as possible because of anticipated difficulties for the teams in obtaining these data for a 12-month period.

#### Diagnosis

Two measures were used: that is, the proportion of new breast cancer patients attending hospital more than twice to achieve a diagnosis (Harcourt *et al*, 1998); and the proportion of new breast cancer patients who had an open biopsy to achieve a diagnosis (Okamoto *et al*, 1998).

#### Therapy

Three measures covering surgery, radiotherapy, and chemotherapy were used, that is, the proportions of new breast cancer patients who received conservation surgery; in whom conservation surgery was followed by radiotherapy; and who were aged under 70 years, node positive, and received adjuvant chemotherapy.

#### Clinical innovation

Three measures were used: that is, who were aged under 70 years, and received induction chemotherapy; entered patients into any of nine open national clinical trials (Stiller, 1989, 1994; routinely measured oestrogen receptor status.

Team member well-being was assessed using a standard psychological measure, the General Health Questionaire GHQ-12 (Goldberg, 1972), widely used for detecting minor psychiatric disorder. It covers feelings of strain, depression, inability to cope, anxiety based on insomnia, lack of confidence, and other psychological problems. This measure had previously been used in the NHS to study cancer clinicians (Ramirez *et al*, 1995, 1996), the mental health of the NHS workforce (Hardy *et al*, 1999), in which the GHQ-12 showed good validity against a psychiatric interview.

### Data analysis

Each part of the inputs–processes–outputs model was tested in turn. For the inputs–processes, and inputs–outputs, parts of the model, the inputs were split into similar types of variable and entered into a stepwise regression analysis to identify possible effects on the outcome. Factors that were identified as significant were entered together in a ‘second-level’ stepwise regression, to identify only genuinely important relationships. A similar procedure was used for the processes–outputs part of the model, except that processes were not used to predict self-reported effectiveness, as previous studies showed that any relationships would be largely affected by common method variance. Other questions involved aggregation of responses from particular occupational groups within teams.

The aggregate measure of clinical performance was derived from the individual clinical measures shown in Table 1

**Table 1:** Clinical data – summary statistics

**Clinical performance variable**	**Mean**	**Standard deviation**	**Minimum**	**Maximum**	**Cutoff^a^ for aggregate clinical performance**
Total number of new cancer patients	182.25	106.52	57	620	Not used
% patients visiting hospital more than twice for diagnostic purposes	10.5%	10.9%	0.0%	52.8%	<8.5%=1
% patients receiving open biopsy	12.0%	11.9%	0.0%	60.2%	<10%=1
% patients receiving lumpectomy	43.4%	17.4%	1.2%	83.7%	>44%=1
% patients receiving radiotherapy after a lumpectomy	72.8%	23.8%	0.0%	100.0%	>78%=1
% patients under 70 (and node+) receiving chemotherapy as part of primary management	64.2%	23.6%	0.0%	100.0%	>70%=1
% patients under 70 receiving induction chemotherapy	17.9%	18.9%	0.0%	80.0%	Not used
Number of clinical trials entered	3.25	2.14	0	9	>3=1
Measure oestrogen receptor status routinely	78% of teams measured oestrogen receptor status routinely				Yes=1

a Based on median values for each variable, all other values=0.

. It was calculated by splitting seven of the clinical data variables dichotomously at their median; teams were assigned a score of 0 if they were in the ‘poorer’ half of the distribution and 1 if they were in the ‘better’ half of the distribution. The overall score was the sum of these, that is, a value between 0 and 7. This score was analysed using both ordinary regression and ordinal logistic regression (not shown): both methods gave the same results, so those for ordinary regression are reported for consistency.

## RESULTS

### Response rates and the samples

Of the sample of 113 breast teams 96 (85%) agreed to participate. For a team to be eligible for inclusion in the analysis, a minimum spread of responses across core disciplines was deemed to be essential. This was defined as at least one breast surgeon and breast nurse together with one member from at least two of the remaining three core disciplines completing a personal team-working questionnaire. A total of 72 (75%) teams fulfilled this requirement (*n*=548 individual responses) and were included in the relevant analyses. However, only 61 (85%) of the 72 teams were also able to provide the required clinical data (*n*=481 individual responses) and only these teams were included in the analyses involving clinical performance.

In the sample of 72 teams, those responding comprised 113 breast surgeons, 122 breast nurses, 108 radiologists, 92 oncologists, and 113 pathologists. The mean age was 45.5 years (s.d. 8.1). Of these, 252 were female (46.5%), and six did not give their gender. The sample of 61 teams was very similar (98 breast surgeons, 104 breast nurses, 97 radiologists, 82 oncologists, and 100 pathologists) with a mean age of 45.4 years (s.d. 8.2). Of these, 220 were female (46.3%) and again six did not give their gender. The spread of disciplines in the teams is shown in Table 2

**Table 2:** Total core membership of breast teams in the sample

	**Numbers and proportions of team members in each core discipline**					
**Number of individuals in that discipline in the team**	**Surgeons**	**Breast nurses**	**Histopathologists**	**Radiologists**	**Clinical oncologists**	**Medical oncologists**
0	0	0	0	0	1 (1%)	51 (71%)
1	17 (24%)	23 (32%)	17 (24%)	23 (32%)	47 (65%)	17 (24%)
2	46 (64%)	27 (38%)	26 (36%)	35 (49%)	18 (25%)	4 (5%)
3	9 (12%)	20 (28%)	25 (35%)	11 (15%)	6 (9%)	0
4	0	2 (2%)	3 (4%)	3 (4%)	0	0
5	0	0	1 (1%)	0	0	0

.

The year in which each team was established (team ‘age’) is shown in Table 3

**Table 3:** ‘Age’ of teams in sample

**Year of formation^a^**	**Number of teams**	
1980–1984	2	0.4 pa^b^
1985–1989	6	1.2 pa
1990–1994	16	3.2 pa
1995–1999	47	9.4 pa
		
Breakdown of 1995–99		
1995	8	
1996	9	
1997	14	
1998	9	
1999	7	

a Year in which regular team meetings began,

b Per annum.

. Most were formed in the 5 years prior to the study, 1997 was the modal year.

### Team inputs and team processes

One team composition factor was a predictor of team processes; professional diversity positively predicted reflexivity (*β*=0.343, *P*=0.009, controlling for team size).

### Team inputs and self-reported effectiveness

A number of team composition factors predicted self-reported effectiveness. There was a consistent and significant difference between disciplines in their perceptions of a number of effectiveness measures, with breast surgeons and breast nurses rating consistently higher than other disciplines in their team, as shown in Table 4

**Table 4:** Effect of discipline on perceived effectiveness

**Discipline**	**Higher rating of their team effectiveness**	**Discipline**	**Lower rating of their team effectiveness**
Breast surgeon	Overall effectiveness	Histopathologist	Overall effectiveness*
	Task orientation		Innovation
	Reflexivity		S-R effectiveness
	Innovation		4* out of 8
	5 out of 8 S-R effectiveness	Radiologist	Overall effectiveness*
Breast Nurse	Overall effectiveness*		S-R effectiveness
	Reflexivity		5* out of 8
	Support for innovation*		
	Innovation*		
	S-R effectiveness		
	All 8 (6 at *)		

All significant at 5% level

* at 1% level. S-R=self-rated.

. Of the significant individual relationships, team size positively predicted accurate and timely diagnosis (β=0.254, *P*=0.034). Greater professional diversity in the team positively predicted effectiveness with respect to clinical audit and research (*β*=0.428, *P*=0.001). The proportion of medical oncologists (note: numbers in this discipline were much lower than others, and biased towards major centres) positively predicted overall effectiveness and effective internal communication (*β*=0.243, *P*=0.044). The proportions of breast surgeons and medical oncologists predicted greater effectiveness of psychosocial support (*β*=0.230, *P*=0.039; *β*=0.297, *P*=0.008). The proportion of radiologists negatively predicted psychosocial support (*β*=0.235, *P*=0.035), and the proportion of clinical oncologists negatively predicted efficient use of resources (*β*=0.241, *P*=0.041).

### Team inputs and clinical performance

In multivariate analysis, two input variables predicted the aggregated measure of clinical performance: the proportion of breast care nurses in the team and workload (caseload per Whole Time Equivalent team member) positively predicted clinical effectiveness (*β*=0.376, *P*=0.003 and *β*=0.331, *P*=0.009, respectively). In univariate analysis, there was a significant correlation (*r*=0.262, *P*=0.045) between new cancer caseload and aggregated clinical performance. The partial correlation, controlling for team size, was 0.319, *P*=0.015. Thus, even when controlling for team size, a larger caseload positively predicted better clinical performance.

Significant relationships were also found between team inputs and individual clinical performance measures, summarised in Table 5

**Table 5:** Summary of relationships between team inputs and individual clinical performance measures

**Input variable**	**Clinical performance**	***β***	***P***
*Team task*			
Higher workload	Greater % patients receiving lumpectomies	0.292	0.029
Greater number of patients seen	More patients entered into clinical trials	0.312	0.018
Greater number of hospitals where breast clinics are held	Greater percentage of patients visiting hospital for diagnosis on more than one occasion	0.416	0.006
	Greater percentage of patients entered into clinical trials	0.323	0.016
Greater number of hospitals where teams are based	Fewer lumpectomies	−0.342	0.011
	More radiotherapy after lumpectomy	0.308	0.012
Greater team age	More radiotherapy after lumpectomy	0.482	<0.001
			
*Team composition*			
More breast care nurses in the team	Fewer open surgical biopsies	−0.333	0.009
More medical oncologists	Fewer lumpectomies	−0.294	0.024
	More radiotherapy after lumpectomy	0.317	0.018
	More patients under 70 years receiving chemotherapy	0.341	0.022
Greater proportion of histopathologists	More open surgical biopsy	0.285	0.024
Greater age diversity	Greater% of patients under 70 years receiving induction chemotherapy	0.324	0.028
Higher mean age of team members	Fewer patients entered in clinical trials	−0.330	0.010

*β*=standardised coefficient in the regression equation; *P*=statistical significance.

.

The frequency of open surgical biopsies was positively predicted by the proportion of histopathologists in the team (*β*=0.285, *P*=0.024), but the number of breast care nurses was associated with fewer open surgical biopsies (*β*=−0.333, *P*=0.009). The number of hospitals where breast clinics were held had a negative impact on the speed of diagnosis, and was positively associated with the percentage of patients visiting hospital for diagnosis on more than one occasion (*β*=0.416, *P*=0.006). However, where breast clinics were held in more hospitals, this was positively associated with the percentage of patients entered into clinical trials (*β*=0.323, *P*=0.016). Recruitment into clinical trials was positively predicted by the number of patients seen (*β*=0.312, *P*=0.018), and negatively predicted by the mean age of team members (*β*=−0.330, *P*=0.010).

The use of lumpectomies was positively predicted by workload (*β*=0.292, *P*=0.029). However, greater number of hospitals where teams were based and more medical oncologists in the team were associated with fewer lumpectomies (*β*=−0.342, *P*=0.011 and *β*=−0.294, *P*=0.024, respectively). The use of radiotherapy after lumpectomy was found to be positively related to the age of the team (*β*=0.482, *P*<0.001), the number of medical oncologists in the team (*β*=0.317, *P*=0.018), and the number of hospitals where teams were based (*β*=0.308, *P*=0.012). Induction chemotherapy was more frequent in teams with a greater age diversity (*β*=0.324, *P*=0.028), and in teams with more medical oncologists.

### Team process and effectiveness

Leadership results are summarised in Table 6

**Table 6:** Relationships between leadership and team effectiveness

**Leadership variable**	**Dependent variable**	***β***	***P***
Having a number of leaders	Participation	0.305	0.016
	Focus on quality	0.374	0.012
	Reflexivity	0.420	0.005
	Innovation (self-rated)	0.224	0.041
	Effectiveness (overall)	0.258	0.018
			
Lack of clarity over leadership	Participation	−0.470	<0.001
	Support for innovation	−0.538	<0.001
	Mean TCI score	−0.573	<0.001
	Clarity of objectives	−0.430	0.004
	Innovation (self-rated)	−0.392	0.001
	Effectiveness (overall)	−0.382	0.001
	Effectiveness – audit/research	−0.511	<0.001
	Efficient use of resources	−0.480	<0.001
	Effective comm. with patients	−0.296	0.012
	Effective internal comm.	−0.431	<0.001
			
Conflict over leadership	Participation	−0.312	0.014
	Support for innovation	−0.515	<0.001
	Mean TCI score	−0.453	0.001
	Effectiveness – audit/research	−0.287	0.007
	Effective internal comm.	−0.367	0.001
			
Having a single, clear leader	Support for innovation	−0.414	0.002
	Mean TCI score	−0.384	0.004
	Effectiveness – audit/research	−0.353	0.002

*β*=standardised coefficient in the regression equation; *P*=statistical significance; TCI=Team Climate Inventory.

. The number of different occupational groups reported by team members as leading team discussion positively predicted team processes and team effectiveness. This dispersed leadership predicted participation, concern for quality and reflexivity, self-rated innovation, and overall effectiveness. Lack of clarity about leadership and conflict over leadership both negatively predicted team processes, participation, support for innovation, and mean team-working score. In addition, lack of clarity about leadership negatively predicted clarity of objectives, overall team effectiveness, efficient use of resources, and effective communication with patients. Conflict over leadership and lack of clarity about leadership were both negative predictors of effective internal communication within the team and effectiveness with respect to audit and research. Having a single, clear leader negatively predicted support for innovation, the mean team-working score, and effectiveness with respect to audit and research.

### Mental health in breast cancer teams

The prevalence of minor psychiatric morbidity among members of breast cancer care teams shown in Table 7

**Table 7:** Comparison of mental health (caseness^a^) between breast teams and the NHS, other health teams, and the population (Mullarky *et al*, 1999)

**Comparison group**	**Caseness^a^ level**
NHS workforce	26.6%
Community mental health teams	25.5%
Secondary-care teams	23.3%
Primary health-care teams	22.2%
British Household Panel Survey	20.6%
Breast cancer teams	15.7% *P*<0.005
	(CI 12.7–18.7%)

a A case is defined as someone who answers in the upper two categories for at least four of the GHQ items. CI is the 95% confidence interval.

was substantially and significantly lower than has been previously observed for people working in other types of health teams, in the NHS workforce generally, and in sample studies of the UK population. Only one measure of team inputs, greater age diversity in the team, predicted better mental health (*β*=0.317, *P*=0.007). This held when age was controlled for.

## DISCUSSION

All three categories, team inputs, processes, and outcomes contained substantial numbers of variables, highlighting the potential for spurious significance results arising from the large numbers of individual comparisons possible within the data. Prior hypotheses were therefore developed about potential relationships within the data, based on studies of teams in other contexts and expert advice about what might be anticipated in breast cancer. This reduced but could not eliminate this problem.

A second concern was attributing causality in significantly correlated relationships within the data, which necessitated caution in interpreting apparently significant findings. For example, predictions involving medical oncology were based on less than a third of teams having a medical oncologist (29% of the 72 sample, 31% of the 61 sample). This distribution was almost certainly not random because of the preponderance of academic departments of medical oncology (mainly in cancer centres).

Four main conclusions were drawn from this study:

- Important relationships existed between team composition and outcome (both perceived team effectiveness and clinical performance).
- Higher breast cancer workload predicted better clinical performance.
- Leadership styles were important to team effectiveness.
- Mental health was substantially better in breast team members than in other NHS settings or the wider population.

### Inputs and effectiveness

The IOG (1996) concluded that there was considerable support for the role of breast nurses, but lacked evidence of their effectiveness. The finding that the proportion of breast nurses strongly predicted (*P*=0.003) aggregated clinical performance in this study has provided important corroboration of their value. It shows that they improved the quality of clinical care, exerting a positive influence on the work of their teams and hence on their medical colleagues. An example was the negative relationship of the number of breast nurses to the proportion of open surgical biopsies. This procedure was once common, but has been gradually replaced by more appropriate and acceptable methods for patients. Breast nurses, whose role includes advocacy of their patient's interests, might have been expected to influence their surgical colleagues and hence accelerate the decline of this procedure. The study results were consistent with this interpretation.

Few relationships were found between team composition and team processes, less than in other types of teams studied (Borrill *et al*, 2000). However, professional diversity in the team and reflexivity were positively related (*P*<0.009), supporting one of the underlying rationales for bringing different occupational groups together in teams. This being that a greater range of knowledge and experience available to the team would promote discussion, opportunities for learning, and lead to improved services. Entry of patients into clinical trials was more common in teams in which the mean age was younger, perhaps reflecting more recent training and professional enthusiasm. Age diversity also had a positive influence on clinical innovation, using induction chemotherapy as a marker.

The length of time a team had worked together was an important influence (*P*=<0.001) on its ability to ensure patients actually received radiotherapy following conservation surgery. This suggests that teams needed time working together if they were to achieve reliable coordination of different elements of service delivery, and that working together clearly improved their teams' clinical performance over time. This is important because of the value of radiotherapy in achieving good local control (Wong and Harris, 2001). The variability in radiotherapy utilisation shown in Table 1 was consistent with evidence from descriptive studies (Sainsbury *et al*, 1995b).

The extent to which teams worked across different hospitals did not have a consistent impact on their quality of care. Holding breast clinics in more hospitals led to more patient visits for diagnostic purposes (*P*=0.006), an indication of poorer quality of care. This might be a consequence of diagnostic services being stretched or disrupted by these arrangements. However, entry into clinical trials was greater when clinics were in more hospitals (*P*=0.016), which may be explained by the nature of the split site arrangements. For example, combining breast services between smaller hospitals and academic hospitals might promote trial entry. Of the hospitals described as the main base for breast teams spanning two or more hospitals, 40% were university hospitals or cancer centres (data not shown). Basing teams in more than one hospital was less satisfactory, with lower rates of conservation surgery (*P*=0.011); however, the likelihood of radiotherapy after lumpectomy was paradoxically greater (*P*=0.012).

### Profession and discipline

Perceptions of team effectiveness were strongly influenced by the profession/discipline of members, and showed a clear pattern. Breast surgeons and breast nurses had a significantly more positive perception of their team's effectiveness across a range of dimensions than the mean. By contrast, histopathologists and radiologists had a consistently more negative perception. Oncologists were not significantly different from the mean. One hypothesis based on their degree of involvement seemed plausible. For breast surgeons and breast nurses, the breast team was central to their work and professional lives. Most oncologists manage patients with a number of cancer types (typically three), involving membership of an identifiable team. Consequently, their commitment to any one team might be expected to be weaker than breast surgeons and nurses who only worked in one. However, the weakest level of commitment to the breast team might be expected among those histopathologists and radiologists who belonged to several other cancer teams as well as having obligations to the running of their departments. This interpretation is consistent with the evidence from this study.

The policy objective of site specialisation among pathologists and radiologists was intended to develop expertise, with higher standards of reporting, and improved diagnostic inputs to clinical decisions (Department of Health, 1995, 2000) and guidance (BASO, 1995; IOG, 1996). However, the requirement for named pathologists/radiologists for each specialist cancer team increased the practical obstacles to full individual participation in any one team. Both disciplines are acknowledged (Department of Health, 2000) to be in short supply. Pressure to site-specialise may have reduced operational flexibility, particularly in smaller departments. Inadequate site specialisation in these disciplines would lead to all or most consultants reporting breast results, and hence being listed as breast team members. Table 2 shows that 40% of teams listed three or more histopathologists, and 19% three or more radiologists, figures suggestive of inadequate site specialisation. Teams with more members in these disciplines were significantly less effective for two measures; more radiologists negatively related to psychosocial support; more pathologists related to more open surgical biopsy.

### Workload, caseload, and clinical performance

This study showed that workload (new cancer annual caseload of the team related to the actual time committed by each breast team member) predicted the quality of clinical care provided by the team as a whole, based on the aggregate measure of clinical performance (*P*=0.009). Notably, it showed that benefit from higher workload was progressive rather than a step effect. Caseload was also significantly correlated in univariate analysis and showed, for a given team size, that higher caseload led to better clinical performance. These data add to other evidence about volume, specialisation and outcome in breast cancer (Sainsbury *et al*, 1995a; Gillis and Hole, 1996). In all, 12 further studies were listed in the evidence review (IOG, 1996), mostly from cancer registries or administrative databases. Univariate analysis also showed that greater numbers of patients was related to greater involvement in clinical trials (*P*=0.018). Overall, this evidence supported national guidance (IOG, 1996) and clinical guidelines (BASO, 1995) that sufficient workload is necessary for breast teams to be viable and effective.

### Leadership and team effectiveness

The term leadership was used to describe leadership within team meetings, and focused on clinical decision-making. The style of leadership was important to the effectiveness of teams. The most effective model, which positively and strongly correlated with five measures (participation, focus on quality, overall effectiveness, self-rated innovation, and reflexivity), was that of a number of leaders within the team, that is plural, democratic, or distributed leadership. This has important implications for breast team development. It also emphasised the importance of the distinction between the (usually) single administrative head of each team, necessary for management, and the shared leadership style within the team which worked best for clinical decision-making.

Lack of clarity or conflict about leadership was strongly and negatively related to effectiveness. This was particularly evident for ‘lack of clarity’, which showed significant negative findings for 10 measures (half at <(*P*=)0.001 level). Conflict was a strong negative predictor across five measures (with *P*-values between <0.001 and 0.014). The alternative of one clear leader had a negative correlation with innovation and effectiveness in audit/research (both *P*-values 0.002), and mean TCI score (*P*=0.004). This suggested that single leaders might be autocratic, in the sense that other viewpoints within the team, which, if expressed, could lead to better decision-making, innovation, and change, did not flourish under this leadership style. Having a single leader was however better for the team than conflict or lack of clarity about leadership.

### Mental health of breast teams members

The results showed psychiatric morbidity significantly below those in previous studies of NHS teams, the NHS workforce, the population as a whole, and particularly in studies of cancer clinicians in the era prior to the widespread adoption of teams in cancer care. Ramirez *et al* (1995) had suggested that there might be latent problems among cancer clinicians of ‘burn-out’ or psychiatric morbidity. For this reason, it was appropriate to include this topic in personal questionnaires. Ramirez *et al* (1995), (1996) had found a prevalence of 27% psychiatric morbidity in 1133 hospital consultants, largely surgeons, radiologists, and oncologists using the same instrument as this study, the GHQ-12, with a prevalence for cancer clinicians only of 28%. The results for breast teams in this sample were very different from those previously described and warrant further study.

There were three possible interpretations for this finding, which were not exclusive. The most likely was that team working was beneficial to the mental health of its members, allowing problems and pressures to be shared, and individuals to be supported by colleagues. Secondly, working in breast cancer may have been a more positive experience relative to other cancers, with a higher disease profile and better prognosis than most solid tumours. The third possibility was selection bias. Could those breast teams (and individuals within them) that participated, and returned their personal questionnaires, be different from the remainder? There seems no obvious reason to attribute different motivations to those completing questionnaires in this study to those responding to Ramirez (Ramirez *et al*, 1995, 1996).

Multidisciplinary teams have been repeatedly identified as central to the delivery of cancer services. They provide the principal mechanism to ensure that the expertise of each relevant discipline and professional group are brought together, contributing to, and participating in, decisions on the management of all patients with a particular cancer type. This study demonstrated that a number of the characteristics of breast teams affected their clinical performance and effectiveness. These findings should facilitate the development of breast teams to maximise their effectiveness. Further studies are required to assess the extent to which these findings might apply more generally to teams managing patients with other cancers, or providing palliative care. The possible beneficial effect on mental well-being warrants further investigation.

## References

[bib1] Adorian D, Silverberg DS, Tomer D, Wamasher Z (1990) Group discussion with the health care team: a method of improving care of hypertension in general practice. J Hum Hypertens 4(3): 265–268 2362258

[bib2] Berino F, Capocaccia R, Esteve J, Gatta G, Hakulinen T, Micheli A, Sant M, Verdecchia A (eds) (1999) Survival of Cancer Patients in Europe: the EUROCARE-2 Study, Vol. 151. Lyon: IARC Scientific Publ. 10619122

[bib3] Berino F, Sant M, Verdecchia A, Capocaccia R, Hakulinen T, Esteve J (eds) (1995) Survival of Cancer Patients in Europe: The EUROCARE Study, Vol. 132. Lyon: IARC Scientific Publ.

[bib4] Borrill CS, West MA, Shapiro D, Rees A (2000) Team working and effectiveness in health care. Br J Health Care Manage 6(8): 364–371

[bib5] Bradley M (1996) The role of the practice pharmacist – the new member of the team. VFM Update Primary Focus Issue 2: 26–27

[bib6] British Association of Surgical Oncology (BASO) (1995) Guidelines for surgeons in the management of symptomatic breast disease in the United Kingdom. Euro J Surg Oncol 21(Suppl A): 1–13 7589617

[bib7] Chouillet AM, Bell CMJ, Hiscox JG (1994) Management of breast cancer in Southeast England. BMJ 308: 168–171 8204162 10.1136/bmj.308.6922.168 PMC2542520

[bib8] Department of Health (1995) Policy Framework for Commissioning Cancer Services: A Report by the Expert Advisory Group on Cancer to the Chief Medical Officers of England and Wales. London: HM Stationary Office

[bib9] Department of Health (2000) The NHS Cancer Plan. London: Department of Health

[bib10] Forrest AP (1986) Breast Cancer Screening: A Report to the Health Ministers of England and Wales. London: HMSO

[bib11] Gillis CR, Hole DJ (1996) Survival outcome of care by specialist surgeons in breast cancer: a study of 3786 patients in the west of Scotland. BMJ 312: 145–148 8563532 10.1136/bmj.312.7024.145 PMC2349835

[bib12] Goldberg DP (1972) The Detection of Minor Psychiatric Illness by Questionnaire. Oxford: Oxford University Press

[bib13] Guzzo RA, Shea GP (1992) Group performance and inter-group relations in organisations. In Handbook of Industrial and Organizational Psychology, Dunnette MD, Hough LM (eds) Vol 3, pp 269–313. Palo Alto, CA: Consulting Psychologists Press

[bib35] Harcourt D, Ambler N, Rumsey N, Cawthorn SJ (1998) Evaluation of a one-stop breast lump clinic: a randomised controlled trial. Breast 7(6): 314–319

[bib14] Hardy GE, Shapiro DA, Haynes CE, Rick JK (1999) Validation of the General Health Questionnaire using a sample of employees from the Health Care Services. Psychol Assess 11: 159–165

[bib15] Hasler J (1994) The Primary Care Team. Royal Society of Medicine Press: London

[bib16] IOG (1996) Cancer Guidance Sub-group of the Clinical Outcomes Group. Improving Outcomes in Breast Cancer, Produced by Department of Health. Manual Cat. Nos. 96 CC0021 & Research Evidence 96 CC0022, July 1996

[bib17] Jansson A, Isacsson A, Lindholm LH (1992) Organisation of health care teams and the population contacts with primary care. Scand J Primary Care 10: 257–265 10.3109/02813439209014071 1480864

[bib18] Kirkhart DG (1995) Sharing care: improving health care, reducing costs. Nurs Manage 26: 6 7770216

[bib36] Landheer ML, Therasse P, van de Velde CJ (2001) The importance of quality assurance in surgical oncology in the treatment of colorectal cancer. In Surgical Oncology Clinics of North America. Vol 10(4): 885–914, Philadelphia: WB Saunders Company 11641097

[bib37] Link JS (2000) History and overview of comprehensive interdisciplinary breast centers. In Surgical Oncology Clinics of North America Vol 9(2): 147–157, Philadelphia: WB Saunders Company 10757839

[bib19] Marsh GN (1991) Caring for larger lists. BMJ 303: 1312–1316 1815559 10.1136/bmj.303.6813.1312 PMC1671410

[bib20] Mullarky S, Wall TD, Warr PB, Clegg CW, Stride CB (1999) Measures of Job Satisfaction, Mental Health and Job-related Well-being. Sheffield: Institute of Work Psychology

[bib21] NHS Executive (1997) Changing the Internal Market EL(97)33: Annex E Investing in Breast Cancer Services. London: Department of Health

[bib22] Norrgren F, Schaller J (1999) Leadership style: its impact on cross-functional product development. J Product Innovation Manage 16: 377–384

[bib38] Okamoto H, Ogawara T, Inoue S, Kobayashi K, Sekikawa T, Matsumoto Y (1998) Clinical management of nonpalpable or small breast masses by fine-needle aspiration biopsy (FNAB) under ultrasound guidance. Journal of Surgical Oncology 67(4): 246–250 9579372 10.1002/(sici)1096-9098(199804)67:4<246::aid-jso7>3.0.co;2-8

[bib23] Ramirez A, Graham J, Richards M, Cull A, Gregory W, Leaning M, Snashall D, Timothy A (1995) Burnout and psychiatric disorder amongst cancer clinicians. Br J Cancer 71(6): 1263–1269 7540037 10.1038/bjc.1995.244 PMC2033827

[bib24] Ramirez A, Graham J, Richards M, Gregory W (1996) Mental health of hospital consultants: the effects of stress and satisfaction at work. Lancet 347(9003): 724–728 8602002 10.1016/s0140-6736(96)90077-x

[bib25] Sainsbury R, Haward B, Rider L, Johnston C, Round C (1995a) Influence of clinician workload and patterns of treatment on survival from breast cancer. Lancet 345: 1265–1270 7746056 10.1016/s0140-6736(95)90924-9

[bib26] Sainsbury R, Rider L, Smith A, Macadam A, The Yorkshire Breast Cancer Group (1995b) Does it matter where you live? Treatment variation for breast cancer in Yorkshire. Br J Cancer 71: 1275–1278 7779723 10.1038/bjc.1995.246 PMC2033815

[bib39] Stiller CA (1989) Survival of patients with cancer. BMJ 299(6707): 1058–1059 2511962 10.1136/bmj.299.6707.1058 PMC1837976

[bib40] Stiller CA (1994) Centralised treatment, entry to trials and survival. British Journal of Cancer 70(2): 352–362 8054285 10.1038/bjc.1994.306 PMC2033490

[bib27] Tannenbaum SI, Salas E, Cannon-Bowers JA (1996) Team building and its influence on team effectiveness: an examination of conceptual and empirical developments. In Issues, Theory and Research in Industrial/Organisational Psychology, Kelly K (ed) pp 503–529, Chichester: Wiley

[bib28] The Macmillan Directory of Breast Cancer Services in the UK: 2nd Edition (1996) Raising Standards in Breast Cancer Care. London: Cancer Relief Macmillan Fund

[bib29] The provision of breast services in the UK: the advantages of specialist breast units. Report of a Working Party of the British Breast Group. February 1995

[bib32] West MA (1996) The Handbook of Work Group Psychology. Chichester: Wiley

[bib30] West MA, Anderson NR (1996) Innovation in top management teams. J Appl Psychol 81(6): 680–693

[bib31] West MA, Borrill CS, Unsworth K (1998) Team effectiveness in organisations. In International Review of Industrial Organisational Psychology, Cooper CL, Robertson IT (eds) Vol 13. Chichester: Wiley and Sons

[bib33] Wong J, Harris J (2001) Importance of local control in breast cancer. Lancet Oncol 2(5): 11–17 11905613 10.1016/S1470-2045(00)00190-X

[bib34] Wood N, Farrow S, Elliott B (1994) A review of primary health care organisation. J Clin Nurs 3(4): 243–250 8055172 10.1111/j.1365-2702.1994.tb00395.x

